# Modeling the Relationship between Safety Climate and Safety Performance in a Developing Construction Industry: A Cross-Cultural Validation Study

**DOI:** 10.3390/ijerph14040351

**Published:** 2017-03-28

**Authors:** Hafiz Zahoor, Albert P. C. Chan, Wahyudi P. Utama, Ran Gao, Irfan Zafar

**Affiliations:** 1 Department of Building and Real Estate, The Hong Kong Polytechnic University, Hung Hom, Kowloon, Hong Kong 999077, China; albert.chan@polyu.edu.hk (A.P.C.C.); wahyudi.utama@connect.polyu.hk (W.P.U.); ran.gao@connect.polyu.hk (R.G.); irfan.zafar@connect.polyu.hk (I.Z.); 2 Department of Construction Engineering and Management, National University of Sciences and Technology, Risalpur Campus, Risalpur 24080, Pakistan

**Keywords:** safety climate, safety performance, modeling, cross-validation, construction industry, Pakistan, developing countries

## Abstract

This study attempts to validate a safety performance (SP) measurement model in the cross-cultural setting of a developing country. In addition, it highlights the variations in investigating the relationship between safety climate (SC) factors and SP indicators. The data were collected from forty under-construction multi-storey building projects in Pakistan. Based on the results of exploratory factor analysis, a SP measurement model was hypothesized. It was tested and validated by conducting confirmatory factor analysis on calibration and validation sub-samples respectively. The study confirmed the significant positive impact of SC on *safety compliance* and *safety participation*, and negative impact on *number of self-reported accidents/injuries*. However, *number of near-misses* could not be retained in the final SP model because it attained a lower standardized path coefficient value. Moreover, instead of *safety participation*, *safety compliance* established a stronger impact on SP. The study uncovered *safety enforcement and promotion* as a novel SC factor, whereas *safety rules and work practices* was identified as the most neglected factor. The study contributed to the body of knowledge by unveiling the deviations in existing dimensions of SC and SP. The refined model is expected to concisely measure the SP in the Pakistani construction industry, however, caution must be exercised while generalizing the study results to other developing countries.

## 1. Introduction

Safety research, spanning over the past 36 years, has gradually progressed from the identification of various safety climate (SC) factors to the investigation of safety outcomes, followed by, establishing the causal relationships between SC and safety performance (SP), and examining the mediating role of various factors such as safety knowledge and motivation [1,2,3,4,5]. Since the pioneering study of Zohar [6], researchers started focusing on the examination of industry and organization-specific SC measures that resulted in the grouping of over fifty conceptual themes related to the construction industry (CI) [3].

Most of the SC studies were conducted in the CI of developed (Western) countries having a homogenous cultural environment such as USA, UK and Canada [7]. However, some of them were also carried out in the Eastern countries such as China, Singapore, Hong Kong and Australia [8,9,10,11,12]. Surprisingly, limited attention was given to the generalization of SC studies across different cultures, languages and regions [13]. Therefore, only a few of the SC studies could be successfully replicated across regions such as Italy [7], China [9], Sweden [14] and Serbia [15]. On the other hand, factor-structure relationships developed by Cheyne et al. [16] for the UK and France could not be cross-validated in the Asian cultural setting of the Malaysian industry [17]. Likewise, in another cross-cultural and cross-language research conducted in USA for Hispanic and non-Hispanic construction workers, non-equivalences were observed in the parameters and error variances of SC factor structure [18].

The inconsistencies observed in the aforesaid cross-validation studies can be attributed to the cultural differences in risk perception, varied social and economic conditions in different regions, preferences for challenging the authority and variations in national safety regulations [3,19]. Realizing these variances, more cross-validation studies are suggested in different regions and cultures [13,19]. This study, therefore, develops an exhaustive approach to investigate the causal relationship between construction SC and SP for developing countries, in general, and Pakistan, in particular. It will disclose the variances in the influence of various SC factors on the indicators of SP. In addition, it is expected to guide the construction stakeholders to measure, monitor and improve the SP in the CI. To the authors’ knowledge, this is the first cross-validation study examining the potential linkages among the constructs of SP measurement model in the cross-cultural setting of the Pakistani CI.

### 1.1. Relationship between SC and SP

SC, being the robust leading indicator of safety outcome [1,5,6,7], has been expansively studied in developed countries at multiple levels such as the team, project, organization, industry and national levels. The most frequently documented SC factors in the CI include: perception of managerial commitment and employees’ involvement in safety, safety communication, safety training, safety systems and procedures, and workers’ attitude to safety and risk [7,10,19,20]. These SC factors have established a significant positive relationship with SP [8,21], however, consensus could not be achieved on the influence of various SC factors on SP in different industries, regions and cultures [5,19].

In the occupational safety research literature, SP is conceptualized as multi-dimensional [22], however, earlier studies used only the number of accidents as an indicator of SP. Later, this indicator was refined as “number of accidents/injuries and near-misses” [23,24]. The advantage of using “near-miss” as a leading SP indicator is that it does not result in any injury or fatality despite the existing potential. It can also warn the management about any potential incident in a proactive way. As accidents/injuries occur infrequently and are not effective indicators of SP, a more refined measure of SP is suggested by the researchers such as “safety behavior” that is measured using safety compliance and safety participation [1,2,4,23,25,26].

Safety compliance comprises of core safety activities that need to be carried out by individuals to maintain a minimum level of safety at the workplace such as wearing personal protective equipment, following safety rules and complying with occupational safety regulations [26]. Safety participation is conceived as an activity that may not directly supplement workplace safety but help in developing an environment that stimulates safety such as voluntarily joining safety training programs and helping coworkers with safety-related issues [26,27]. According to DeArmond et al. [22], safety participation refers to the behaviors that are voluntary in nature, while safety compliance refers to the behaviors that are compulsory. It is worth mentioning that there is no single measure of SP that can be said to be superior to others, however, safety participation has developed a stronger positive relationship with SP as compared to safety compliance [2].

### 1.2. Construction Safety in Developing Countries

Compared to the past, the current decade is witnessing a rise in the infrastructure growth in developing countries such as Pakistan, where the share of CI in national GDP (Gross Domestic Product) [28] has increased from 2.3% in 2012–2013 to 2.44% in 2014–2015; reflecting its consistent contribution in the country’s economic development. Although no significant increase was noticed in the employment rate of CI during the last 6 years, it suffered an increase in the percentage of injuries from 14.1% in 2013–2014 to 16.3% in 2014–2015 ([29], p. 38). It was, therefore, ranked as the second most injury-prone industry in Pakistan, after agriculture. These accidents also resulted in cost overruns, construction delays, extended non-appearance of workers at worksites, lower productivity and conflicts between the key stakeholders in the CI [30,31]. More specifically, the construction companies working on building projects, despite following various safety management systems, are persistently suffering from fatal accidents, mostly due to *fall from height* and *electrocution* [32,33]. Such a situation warrants further examination of safety measures on building projects.

## 2. Research Methods

### 2.1. Questionnaire Design

A survey questionnaire enables the researcher to reply the specified questions by testing the hypotheses and evaluating the results. Thus, a validated questionnaire with some modifications was adopted for this study to collect and analyze the SC and SP data [23]. It was presented in English as well as in Urdu (the national language of Pakistan). The Urdu version was developed especially for the frontline workers who were unable to read and/or understand the English language. The Urdu version was re-translated into English and compared with the original one, in order to observe any change in the meaning of each statement [7]. The questionnaire was also checked for its consistency and reliability by two academics and two industry practitioners. Both the English and Urdu versions are available from the authors on request.

The finalized questionnaire consisted of three parts: personal attributes (six questions), SC (24 statements), and SP (three broad indicators with a total of 10 statements). In the first part, respondents were asked to give the information regarding their age, education level, their direct employer/organization, working level, length of service with the present employer and work experience in the CI [34]. Second and third parts of the questionnaire are explained in the following sub-sections.

#### 2.1.1. Measurement of SC

Most of the SC statements were primarily extracted from the validated 38-item survey questionnaire of the Occupational Safety and Health Council of Hong Kong [10,35,36]. The primary reason for adopting this questionnaire was that it had already been validated for the building projects [10]. Besides, this study’s sample was quite similar to Hong Kong CI as it was collected from the tall building projects in Pakistan where safety regulations are implemented rigorously.

Considering the importance given to regional and cultural values in past studies [9,17], and in the light of experts’ opinion and literature review, some modifications and additions were made in the adopted questionnaire. Respondents were asked to give their level of agreement on a 5-point Likert scale, with 1 being *strongly disagree* to 5 being *strongly agree* [8,9,21,34,37,38]. The detailed development and validation of the 24-item SC scale along with its applicability to Pakistani CI is reported in Zahoor et al. [36], in which twenty-four SC statements were clustered into four factors (Table 1): SCF1—Management commitment and employees’ involvement in health and safety (MC&EI); SCF2—Safety enforcement and promotion (SE&P); SCF3—Applicability of safety rules and safe work practices (SR&WP); and SCF4—Safety consciousness and responsibility (SC&R).

#### 2.1.2. Measurement of SP

SP was measured using three broad indicators of safety compliance, safety participation and “number of self-reported accidents/injuries and near-misses”, as shown in Table 2. Safety compliance was measured in terms of the percentage of time (on a scale of 0%–100%) the safety instructions/procedures were followed on construction site by the worker, his co-workers working in the same team, and all other workers in the company.

The first two questions were adopted from the studies of Mohamed [21] and Hon et al. [23], while the third question was adopted from Zhou et al. [38]. For measuring the safety participation, respondents were asked three questions to assess the existence of an environment that supports the voluntary participation of the employees in safety promotion and implementation. All the three statements were adopted from the study of Neal and Griffin [26] and they were measured on a 5-point Likert scale (ranging from *no participation* to *daily participation*). As the reliable accident statistics were not available, self-reported accident statistics were collected. The ‘number of self-reported accidents/injuries and near-misses’ suffered during the last twelve months, were measured using four questions [23] on a 5-point Likert scale (ranging from *no accidents* to *over five accidents*). Questions were set in an ascending order of injury severity such as near-miss occurrences, injuries not needing absence from work, injuries needing absence from work for not more than three successive days, and injuries requiring absence from work for more than three successive days [23].

### 2.2. Data Collection

#### 2.2.1. Sample Size

A sample size of 200 can guarantee the reliable results if the data set is to be analyzed using confirmatory factor analysis (CFA) [39,40]. For this study, the data were collected from forty under-construction multi-storey building projects in Pakistan (at least 70 m high) during the period from March to June 2015. These projects were spread over six major cities: Karachi (28), Lahore (7), Islamabad/Rawalpindi (3), Faisalabad (1) and Hyderabad (1). To reduce the respondent’s bias, the responses were collected from all types of stakeholders such as clients, consultants, main contractors and subcontractors. On most of the sites, contents of the questionnaire were explained to the respondents. However, at few of the sites, managers were briefed to further explain to their employees the intended benefits of this survey. The confidentiality was also assured to the respondents, and they were at liberty to decline to the questionnaire filling request. A total of 900 survey questionnaires were circulated however only 454 responses could be collected with a response rate of 50.4%. After the imputation of missing values and the dropping of 28 incomplete and unengaged responses, 426 questionnaires were shortlisted for further analysis.

#### 2.2.2. Demographics

The respondents’ distribution is tabulated in Table 3 as per their age, education level, working level, type of employer, and work experience in the current company and industry. Almost all the supervisors and safety officials had a diploma level certification in civil and occupation safety and health respectively. However, workers were mostly uneducated and working without any safety training. Notably, none of the safety officers possessed a university degree in occupational safety and health. Even the safety directors possessed only a national/international level certification in safety, in addition to their professional qualification. Interestingly, young engineers and workers were found more cooperative in giving their feedback to the survey questionnaire as compared to the experienced employees. This fact is evident from the statistics as 68.5% of the respondents were aged below 40 years and over 50% of the respondents had a work experience of fewer than 10 years. Approximately, 52% of the respondents belonged to main and subcontractors. Likewise, over 90% of the respondents had worked with their current employer for a maximum period of 5 years; thus depicting a trend of frequently changing the employer.

## 3. Data Analysis

To start with the analysis, responses to the negatively worded SC statements (SC4, SC11, SC17, SC23, SC26, SC29), collected through a 5-point Likert scale, were reversed. Thus, the values of 1, 2, 4 and 5 were converted into 5, 4, 2 and 1, respectively. The data were randomly split into calibration and validation sub-samples, such that each sub-sample had 213 responses. To identify the SC factors and SP indicators, the statistical technique of exploratory factor analysis (EFA) was conducted on calibration sub-sample using the Statistical Package for the Social Sciences (SPSS ver. 19.0) [36]. Then, a conceptual model showing the causal relationship between the identified SC factors (constructs) and the indicators of SP was hypothesized. It was empirically tested and validated using structural equation modeling (SEM) on calibration and validation sub-samples respectively. This analysis elucidated the association between different variables of the hypothesized model. The details of the data analysis are explained in following sub-sections.

### 3.1. Exploratory Factors Analysis

#### 3.1.1. Data Suitability for Factor Analysis

The data normality was checked using Shapiro-Wilk normality test. To check the data suitability for factor analysis, two tests were conducted on calibration sub-sample: significance value of the Bartlett test of Sphericity <0.05 [41,42], and Kaiser-Meyer-Olkin (KMO) value >0.5 [39,43]. However, a KMO value of 0.8 and above is considered more suitable [44,45]. The data set was observed to have substantial correlations among some of its observed variables; thus verifying the likelihood of correlation matrix not to be an identity matrix [10].

#### 3.1.2. Extraction of SC Factors and SP Indicators

EFA is generally conducted for the data reduction, and to ascertain the factor-structure of a scale and test its reliability [44]. In this study, Principal Component Analysis, which is one of the most commonly used EFA techniques, was applied on calibration sub-sample to extract the factors of SC and SP. The data set was rotated using promax rotation method with Kaiser Normalization. Criteria used to enhance the factor interpretability included: (1) Eigenvalues >1 [39,46]; (2) communalities >0.4 [47]; (3) factor loadings >0.5 [39,41,48]; (4) difference between the cross loadings >0.2 [39]; and (5) Cronbach’s coefficient alpha >0.7 [10,17,45,46]. Another criterion to stop the factor rotation was to achieve 60% of the cumulative variance [49]. In addition, it was ensured to retain at least three items in each extracted factor for achieving an acceptable reliability level [46].

To measure the reliability and internal consistency of the extracted factor-structure, Cronbach’s coefficient alpha value was calculated for each extracted factor and for the complete data set. Generally, Cronbach’s alpha value of 0.7 and above is considered acceptable [50]. However, a value of 0.6 and above can be accepted in case of a newly developed scale [21,39] or if the factor entails a fewer number of observed variables [51].

### 3.2. Development of Research Hypotheses

**Figure 1:**
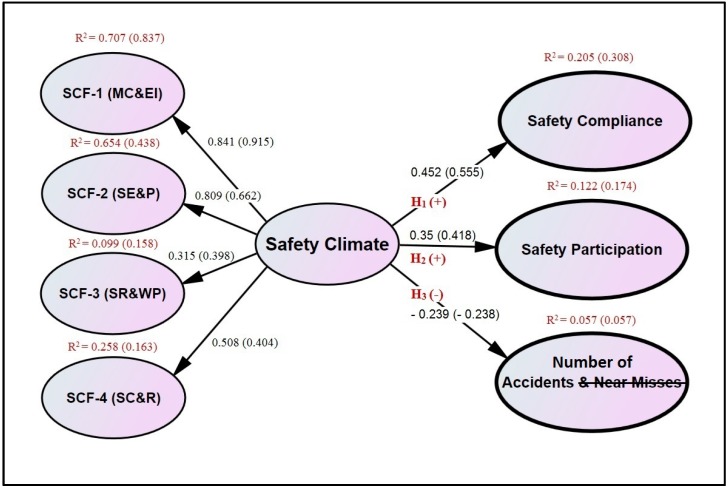
Hypothesized model showing the relationship between SC and SP.

Based on the literature review and results of EFA, a SEM model was hypothesized for this study as shown in Figure 1. The model was tested using Analysis of Moment Structures (AMOS ver. 20.0, IBM, New York, NY, USA), because: (1) it can handle the non-normal data through the maximum likelihood method of estimation, which is the most frequently used and robust estimation method for measuring the structural paths coefficients [52]; (2) its drawing tools are convenient to draw the path diagrams of a model [52,53]; and (3) it can simultaneously compute the explicit estimates of the standardized path coefficients, squared multiple correlations, and error variances for the first and second-order latent variables [54]. The following four hypotheses were established in the posited model, to examine and cross-validate the influence of SC factors on SP indicators in the construction of building projects in Pakistan:

**Hypothesis-1** **(H_1_):** SC has a significant positive relationship with safety compliance.

**Hypothesis-2** **(H_2_):** SC has a significant positive relationship with safety participation.

**Hypothesis-3** **(H_3_):** SC has a significant negative relationship with self-reported accidents/injuries and near-misses.

**Hypothesis-4** **(H_4_):** Safety participation has a stronger positive relationship with SP than safety compliance.

### 3.3. Hypotheses Testing

#### 3.3.1. Model Specifications

**Figure 2:**
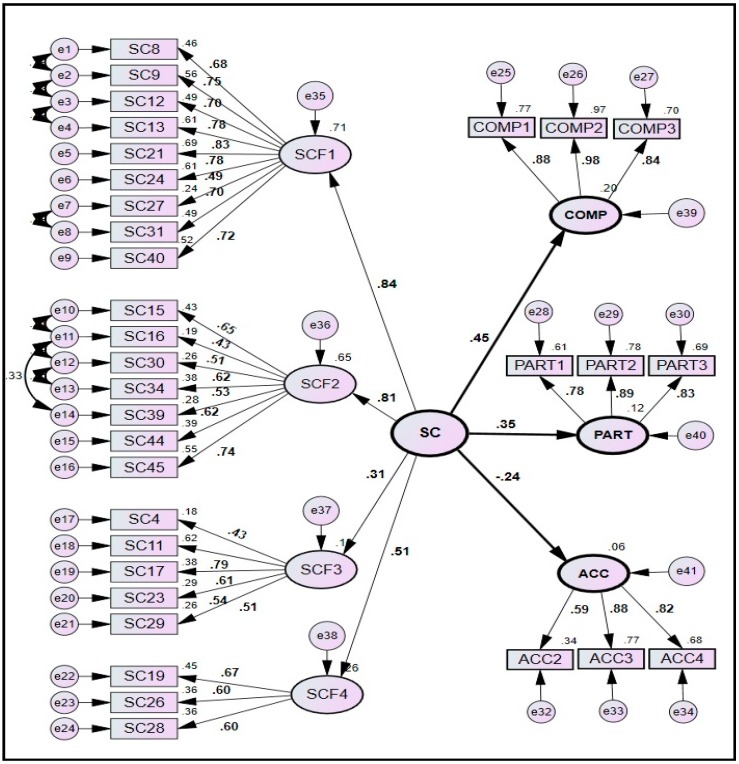
SEM model for calibration sub-sample.

The structural model as shown in Figure 1 displays the causal relationship among the four constructs of SC and three indicators of SP including safety compliance, safety participation, and “number of self-reported accidents/injuries and near-misses”. The hypothesized model also contains four measurement models as shown in Figure 2. The first measurement model determines the SC and it comprises of one second-order and four first-order latent variables, and 24 observed variables. The other three measurement models show the relationship of three indicators of SP with their respective observed variables.

#### 3.3.2. Model Evaluation Using Calibration Sub-Sample

The hypothesized model was evaluated for achieving the desirable model-fit. Criteria initially adopted to enhance the model-fit include: (1) deleting the variables in the regression estimates which could not achieve the significance of 0.05 and below; and (2) deleting the observed variables having a standardized path coefficient and squared multiple correlation of less than 0.5 and 0.25 respectively [39,40]. It was followed by identifying large modification indices that depict the existence of covariance among the error variables [55]. Accordingly, few correlations were drawn among the residuals of observed variables within each factor that resulted in enhancing the model-fit to an extent.

In the next step, model-fit was assessed using the three most common goodness-of-fit (GOF) indices [20,46,50,52,55,56,57]: (1) *parsimonious fit*; the ratio between chi-square and degree of freedom (Chi-sq/df) to be less than 2 for a good model; (2) *absolute fit*; root-mean-square error of approximation (RMSEA) value to range between 0.05 and 0.08, P-close to be less than 0.05, while GOF index (GFI) and adjusted GOF index (AGFI) to range between 0.5 and 1, with 1 representing the perfect fit, however, a value of 0.8 and above is preferred for AGFI, and (3) *incremental fit*; comparative fit index (CFI) to be more than 0.9 preferably [56].

Lastly, to confirm the high predictability and practical value of the developed model, it was tested for achieving the acceptable level of composite reliability, and convergent and discriminant validities.

#### 3.3.3. Composite Reliability

*Composite reliability (CR)*, also known as construct reliability, tests the precision or consistency of a measure [50,58]. The least acceptable value of CR is recommended as 0.7 by Hair et al. [39] and 0.6 by Awang [52]. It was measured using Equation (1) ([52], p. 63):

CR = SSI/(SSI + SEV)(1)

where, SSI is the square of the sum of all factor loadings of a construct, SEV is the sum of all error variances of a construct, and error variance is equal to one minus squared multiple correlation.

#### 3.3.4. Convergent and Discriminant Validities

*Convergent validity* is achieved if the significance of each regression weight is less than 0.05, and all values of standardized regression weights and squared multiple correlations are over 0.5 and 0.25 respectively [56]. Moreover, average variance extracted (AVE) of each construct should be higher than 0.5 but lower than CR of that construct [52].

*Discriminant validity* is achieved if: (1) AVE of a particular construct is greater than the highest squared factor correlation of that construct i.e., maximum shared variance (MSV) [10,56]; (2) AVE is greater than the average shared variance (ASV); (3) square root of AVE of a particular construct is greater than the squared factor correlation among the same construct and other constructs [52,56]; and (4) correlation between exogenous constructs is less than 0.85; thus confirming the non-existence of multicollinearity in the data set [52]. ASV is equal to the mean of squared correlation values of a construct with all other constructs, MSV is the maximum value of the squared correlations of a construct with all other constructs, while AVE is equal to the average of all squared factor loadings of a construct [52].

### 3.4. Model Validation Using Validation Sub-Sample

To validate the results obtained through calibration sub-sample, CFA was repeated on validation sub-sample, and then the values of standardized path coefficients and model-fit indices of both the sub-samples were compared.

## 4. Results

### 4.1. Data Normality and Suitability for Factor Analysis

The results of Shapiro-Wilk normality test pointed out that the significance value of each observed variable in the data set was less than 0.05; indicating that calibration sub-sample is not normally distributed and requires non-parametric tests for further analysis [59]. Significance values of 0.001 for the Bartlett test of Sphericity (Table 4) state the existence of correlations among some of the variables [10]. In addition, it was confirmed by the existence of numerous coefficient values of 0.3 and above in the correlation matrix [49]. Similarly, KMO values for SC (0.848) and SP (0.721) indicate the existence of a marvelous level of sampling adequacy. Hence, the data were found suitable for EFA.

### 4.2. Descriptive Statistics

Table 5 shows the mean, standard deviation and correlation values of the latent variables of SC and SP. As expected, the variable of “number of self-reported accidents/injuries and near-misses (ACC)” is negatively related to all other latent variables. Noticeably, SC factors of SCF2 and SCF3 are observed to be marginally negatively related to each other (−0.035).

### 4.3. EFA for Calibration Sub-Sample

#### 4.3.1. SC Factors

The results of EFA for SC factors are tabulated in Table 1. Except for SC45, all the observed variables have achieved the desirable values of factor loadings and communalities. SC45 was, however, retained in the model as its factor loading (0.495) was marginally below the threshold of 0.5. The values of Cronbach’s coefficient alpha for all the four SC factors (SCF1, SCF2, SCF3, SCF4) were above the threshold of 0.6. Moreover, the derived four-factor structure attained a reasonable level of cumulative variance (56.18%) and each SC factor comprised of at least three items; thereby achieving an acceptable level of reliability. Cronbach’s coefficient alpha value for the complete data set was achieved as 0.885, whereas for all the extracted SC factors, it ranged from 0.648 to 0.908, thus achieving an excellent internal consistency and reliability.

#### 4.3.2. SP Indicators

EFA has extracted three indicators of SP including safety compliance, safety participation, and “number of accidents/injuries and near-misses” (Table 2). Though SP measurement scale achieved a reasonable cumulative variance of 73.968%, it could only attain an overall Cronbach’s coefficient alpha value of 0.68. All the observed variables attained the desirable values of factor loadings (>0.5) and communalities (>0.4) except the communality value for ACC1 (0.345). Thus, ACC1 (depicting the near-misses) was deleted from SP measurement scale. It has increased: (1) Cronbach’s coefficient alpha value for the overall scale from 0.68 to 0.703; (2) Cronbach’s coefficient alpha value for the factor of ACC from 0.732 to 0.79; (3) cumulative variance from 73.968% to 79.566%; and (4) mean value from 2.674 to 2.711. As a result, all the SP indicators achieved excellent internal consistency and reliability.

### 4.4. SEM Results

#### 4.4.1. Model Evaluation and Validation Using CFA

**Figure 3:**
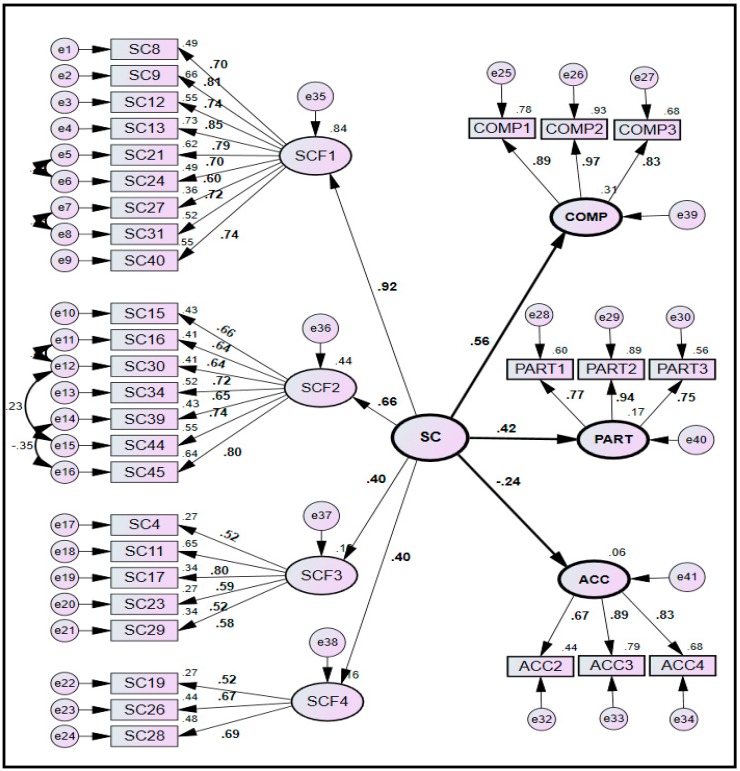
SEM model for validation sub-sample.

The hypothesized model was initially tested using calibration sub-sample. The analysis was then repeated using validation sub-sample, followed by comparing the results of both the sub-samples. The structural and measurement models for both the sub-samples are drawn in Figure 2 and Figure 3. A careful analysis of the measurement models for both the sub-samples revealed that almost all the paths between the first-order latent variables and their respective observed variables were significant as they achieved the desired values of standardized path coefficients (>0.5) and squared multiple correlations (>0.25), except the three paths in calibration sub-sample. These three paths were related to the observed variables of SC4, SC16 and SC27 that could attain marginally lower values of standardized path coefficients as 0.430, 0.431 and 0.491 respectively (Figure 2). However, they were not removed from the model as their deletion could not improve the model-fit significantly.

Furthermore, they achieved the acceptable values of path coefficients in the validation sub-sample (Figure 3). In both the sub-samples, the four first-order latent variables of SC (SCF1, SCF2, SCF3 and SCF4) were observed to significantly influence the three indicators of SP.

#### 4.4.2. Comparison of Model-Fit Indices

The values of three goodness-of-fit (GOF) indices for both the sub-samples are tabulated in Table 6. The calibration sub-sample could achieve the desirable model-fit (Chi-sq/df = 1.999, RMSEA = 0.069, P-close = 0.001, GFI = 0.788, AGFI = 0.753, CFI = 0.858) after deleting the observed variable of ACC1 (having a relatively lower standardized path coefficient of 0.37) and correlating 8 error variables (having higher values of modification indices). These correlations are displayed in Figure 2 by drawing them among the residuals of observed variables. The validation sub-sample also achieved the desirable model-fit as shown in Table 6 (Chi-sq/df = 1.984, RMSEA = 0.068, P-close = 0.001, GFI = 0.778, AGFI = 0.742, CFI = 0.872).

#### 4.4.3. Composite Reliability and Validity

The model has achieved the excellent level of composite reliability and validity for calibration sub-sample (Table 7). All the CR values were greater than 0.6 depicting an excellent level of construct reliability. *Convergent validity* was achieved as: (1) regression weights of all the variables were significant (*p* < 0.05); (2) all the standardized regression weights and squared multiple correlations for the measurements models were above the threshold of 0.5 and 0.25 respectively (except for SC4, SC16 and SC27 in calibration sub-sample only) as shown in Figure 2 and Figure 3; and (3) CR value of each SC factor was higher than AVE of that factor, however, some of the values of AVE were observed to be not higher than 0.5. *Discriminant validity* was achieved as: (1) AVE of each construct (except SCF2) was greater than its MSV and ASV; (2) square root of AVE of a particular construct was greater than the squared factor correlation between that construct and other constructs; and (3) non-existence of multicollinearity was verified as the maximum correlation between the exogenous constructs (0.628) was less than 0.85 as shown in Table 5.

#### 4.4.4. Hypotheses Testing

The postulated model (Figure 1) confirmed the first two hypotheses of H_1_ and H_2_. The relationship between SC and safety compliance (H_1_) was observed to be significantly positive. Standardized path coefficient values of 0.452 and 0.555 for calibration and validation sub-samples respectively depict that when SC goes up by one standard deviation, safety compliance goes up by 0.45–0.55 standard deviation. Similarly, the squared multiple correlations of 0.205 and 0.308 for both the sub-samples reveal that the predictors of safety compliance can explain 20.5% and 30.8% of its variance. The relationship between SC and safety participation (H_2_) was also significantly positive having the standardized path coefficients of 0.35 and 0.418, and squared multiple correlations of 0.122 and 0.174 for both the sub-samples.

H_3_ is partially accepted as the “number of near-misses (ACC1)” could not be retained in the hypothesized model. However, the relationship between SC and the “number of self-reported accidents/injuries” (H_3_) was found to be significantly negative having the standardized path coefficient of −0.239 (−0.238) and squared multiple correlations of 0.057 (0.057) for both the sub-samples. This implies that when SC goes up by one standard deviation, the number of self-reported accidents/injuries goes down by 0.239 standard deviations. Similarly, the predictors of the number of self-reported accidents/injuries can explain 5.7% of its variance.

H_4_ is rejected as safety compliance has developed the stronger influence (0.452) on SC than safety participation (0.35). It can also be inferred that among the three indicators of SP, safety compliance (0.452) and the number of self-reported accidents/injuries (−0.239) have the strongest and the weakest influence on SC respectively.

## 5. Discussion

The study encompasses a systematic hybrid approach integrating the statistical techniques of EFA and CFA to validate the SP measurement model of Hong Kong construction industry in the cross-cultural setting of a developing country. In addition, it reveals the variances in the causal associations between SC factors and SP indicators. The developed model comprises of 24 items of SC grouped into four factors/constructs, and nine items of SP grouped into three indicators/constructs. The model was tested and validated using calibration and validation sub-samples (*n* = 213 each) respectively. The developed model achieved the desired GOF indices, an acceptable degree of composite reliability, and convergent and discriminate validities. Resonating with the results of Hon et al. [23], this study has confirmed the significant positive impact of SC on both the safety compliance and safety participation, and significant negative impact on the number of self-reported accidents/injuries.

Considering the importance given to near-misses in previous studies for forecasting the potential accidents/injuries, the “number of near-misses (ACC1)” was initially included in the hypothesized model as one of the observed variables to measure the SP indicator of ‘number of self-reported accidents/injuries and near-misses (ACC)’ [23]. Notwithstanding, ACC1 could not be retained in the final SEM model due to its lower standardized path coefficient (0.37). Consequently, the related SP indicator was renamed as “number of self-reported accidents/injuries”. Its deletion has not only enhanced the Cronbach’s coefficient alpha value of SP scale from 0.68 to 0.703 (Table 2) but resulted in achieving the desired GOF indices for SEM model. Given the above, it is suggested to further investigate the applicability of observed variable of ACC1 in measuring the SP in other regions and cultures, so as to eventually include or exclude it from SP measurement model.

The findings have deviated, to an extent, from the past studies conducted in different regions. For instance, the SP should be immensely influenced by safety participation than the safety compliance [1,2,60]; however, this perception was confronted in this study, as safety compliance has established a stronger positive impact (0.452) on SP than the safety participation (0.35). Moreover, “number of accidents/injuries” was quantified to have the weakest influence (−0.239) on SC among all the three SP indicators. It is, however, inconsistent with the findings of Hon et al. [23] that could develop the weakest influence of safety participation on SC. These variances in the impact of various SP indicators confirm the necessity of investigating the safety behavior in the cross-cultural and cross-regional environment, especially in the developing countries.

The study has evaluated the relationships between each factor of SC and perceived SP. As presented in Figure 1, SC factor of MC&EI has developed the strongest relationship (0.841) with perceived SP, followed by SE&P (0.809), SC&R (0.508) and SR&WP (0.315). This finding is almost in line with the study of Choudhry et al. [8]. However, it contrasts with the study of Hon et al. [23] that has derived a 3-factor structure, and developed the strongest relationship of perceived SP with the factor of SR&WP as compared to MC&EI. Moreover, the study has identified SE&P as a novel and second most influential SC factor. In the past studies [9,10,14,35,36], SC statements related to this factor were generally distributed among other factors such as management commitment, safety regulations and safety promotion. However, two recent studies identified nearly similar SC factors, namely ‘supervisory care promotion’ and “positive feedback and safety recognition” [61,62]. The emergence of SE&P as a new SC factor also verifies the influence of regional and cultural values on SC. Hence, a SC scale must be validated in the country of intended use [17].

Considering the path coefficients of all SC factors (Figure 2) and their respective percentage of variances (Table 3), it can be concluded that a focus on the factors of MC&EI and SE&P can have a greater impact on SP. Similarly, relatively lower mean values of the factors of MC&EI and SR&WP demand stakeholder’s special attention (Table 1 and Table 5). Despite having a relatively lower mean value, the factor of MC&EI achieved the strongest impact on SC (0.841); thus signifying that a little attention by the higher management will enhance the SP enormously. It can be supplemented with an enhanced employees’ involvement through various incentive schemes and by inculcating the sense of responsibility among its employees [63]. The corresponding variables of this factor can also be considered for potential improvements. For instance; SC21, measuring the safety communication between the higher management and the workers, achieved the highest path coefficient of 0.832 (mean = 2.81) among all the observed variables of MC&EI factor (Figure 2). It indicates that a focus on SC21 can greatly impact the factor of MC&EI, and the overall SC and SP. This factor can also be improved by ensuring that people always wear their PPE, and by providing sufficient resources, equipment and safety training to the workers [23].

Similarly, the lowest mean value of SR&WP factor (2.42) speaks of the appalling situation in the CI of Pakistan where implementation of appropriate safety rules and regulations is lacking and unsafe work practices are not uncommon. One of the main reasons for the lack of compliance to safety instructions is that employees do not see the utility of these safety rules and procedures [60]. The respondents had also expressed their concern that some safety rules and work procedures are obsolete and incompatible to the site constrictions [64]. Thus, it is vital for the organizations to effectively communicate the importance of safety rules to their employees. In addition, there is a need to develop and implement the standardized safety rules and regulations that commensurate with the rapid advancement in the construction methods [4,65].

The unsuccessful cross-validation of Hon et al. [23] in Pakistani CI and deviation in the existing factor structure are in line with past two studies [17,18], as these studies could not be cross-validated in the developing country of Malaysia and for non-Hispanic construction workers in USA. The variances observed in the cross-validation studies can be attributed to the differences in the: workers’ safety awareness, level of education and safety training, job insecurity, competence of higher management and supervisors, subcontracting practices, effectiveness of the safety regulatory authority, and most importantly, workers propensity towards safety compliance and their risk taking behaviour [17,18,37,66,67]. Hence, similar SC factors should not be expected in different countries as well as industries.

### 5.1. Significance and Practical Implications

The study has several theoretical and practical contributions. First, it contributes to the body of knowledge by opposing the prevailing perception that safety participation, compared to safety compliance, has a stronger influence on SP; Second, it discusses the differences observed in the impact of various SC factors on SP in a developing country; Third, it has discovered SE&P as a novel SC factor comprising of seven SC items; this factor might have been split among other SC factors in previous studies. Fourth, although this study was conducted in Pakistani CI, the developed model is expected to measure the SP in developing countries sharing similar work environment. Fifth, an in-depth understanding of the significant SC factors and their respective dimensions may help the key stakeholders in making their construction sites safer. Finally, a comprehensive methodology was presented that can be replicated in other industries and regions.

### 5.2. Limitations and Future Directions

First, the cross-sectional design of the study precludes any conclusion regarding the causality. The second limitation concerns the use of self-reported SP data collected from the building projects in Pakistan; hence, caution must be exercised while generalizing the study results to other developing countries. The results were also dependent on the respondent’s candid opinion and the surveys were not personally administered at some of the construction sites. Nevertheless, this research offers a valuable starting point for future safety interventions in developing countries. As the employees’ perceptions can change over time, a longitudinal study can examine the steadiness of the developed model for developing countries. Realizing the varied influence of cultural and regional aspects and the discovery of SE&P as a novel SC factor, potential differences in safety related perceptions need to be further investigated in other developing regions and cultures [7]. Besides, a multi-level investigation of SC factors is suggested so that the safety behaviour of those employees can be enhanced who are engaged for the subcontracted works in the Pakistani CI [37].

## 6. Conclusions

This study has hypothesized and cross-validated the complex associations of three indicators of SP with twenty-four observed variables and four first-order latent factors of SC in a developing region. It is the first study of its kind that has tested an existing model of SC and SP in the CI of Pakistan and reported the inconsistencies in the interrelationships among the model constructs. A developed questionnaire was used to gather the information (*n* = 426) on variables of interest from forty under-construction multi-storey building projects. EFA was carried out on calibration sub-sample to identify the factors of SC and SP. The extracted SC factors include MC&EI, SE&P, SR&WP and SC&R whereas SP indicators include safety compliance, safety participation and “number of self-reported accidents/injuries”. To test and validate the hypothesized SP measurement model, CFA was conducted on both the calibration and validation sub-samples using AMOS. CFA results offered a perfect understanding of the causal relationship among various constructs of SC and SP. As the developed model achieved the desired GOF indices, construct reliability, and convergent and divergent validities, it is expected to concisely measure the construction SP in the developing countries sharing similar work environment.

The study has confirmed the initial three hypotheses whereas the fourth hypothesis was rejected. It has developed a significant positive impact of SC on both the safety compliance and safety participation, and significant negative impact on the “number of self-reported accidents/injuries” (excluding the near-misses). The “number of near-misses” could not be retained in SP measurement model due to a relatively lower value of standardized path coefficient. In contrast with previous studies, safety compliance has established the strongest positive impact on SP than the safety participation and “number of accidents/injuries”. Similarly, SC factor of MC&EI developed the strongest relationship with perceived SP, followed by SE&P, SC&R and SR&WP. The lowest mean value of SR&WP painted the unpleasant enactment of safety rules and procedures in the CI of Pakistan. Furthermore, the study disclosed SE&P as a novel and second most influential SC factor.

Given the above, construction stakeholders are suggested to focus more on safety compliance for achieving an enhanced SP. In addition, employees need to be motivated to comply with obligatory safe procedures and work practices. Such efforts can be supplemented by inculcating a higher degree of self-motivation among the employees through various incentives to enhance their safety participation. Though safety participation will not contribute directly to personal safety, it will nurture the culture of voluntary participation in the organization. Moreover, there is a need to focus on the factors of MC&EI and SE&P as they can significantly enhance the SP. It is suggested to develop, implement and regularly update the standardized safety rules and regulations matching the rapid technological advancement in the CI.

## Figures and Tables

**Table 1:** Principal component analysis to obtain four-factor structure of SC using calibration sub-sample.

Item No.	SC Statement	Factor Loading
**SCF1—Management commitment and employees’ involvement to health and safety (MC&EI)**		
	(Mean = 2.83, Eigenvalue = 7.168, Variance = 29.868%, Cronbach’s coefficient alpha = 0.908)	
SC8	Company really cares about the health & safety of the people who work here.	0.669
SC9	Adequate health & safety training is given by the company to perform the job safely.	0.687
SC12	People here always wear their personal protective equipment when they are supposed to.	0.836
SC13	All the people who work in my team are fully committed to health & safety.	0.799
SC21	There is always good communication here between management and workers about health & safety issues.	0.824
SC24	Sufficient resources are available for health and safety here.	0.698
SC27	Time pressures for completing the jobs are reasonable.	0.705
SC31	My workmates would react strongly against people who break health & safety procedures.	0.776
SC40	Working with defective equipment is not at all allowed.	0.875
**SCF2—Safety enforcement and promotion (SE&P)**		
	(Mean = 3.46, Eigenvalue = 3.095, Variance = 12.895%, Cronbach’s coefficient alpha = 0.818)	
SC15	The company/management encourages suggestions/feedback from the employees, on how to improve health & safety.	0.516
SC16	There is always good preparedness for emergency here.	0.787
SC30	Accidents which happen here are always reported.	0.768
SC34	Management always motivates and praises the employees for working safely.	0.779
SC39	Safety posters and publications are effectively used for safety awareness.	0.673
SC44	Necessary precautions are taken against fall protection.	0.507
SC45	Supervisors carry out the job hazard analysis before start of each activity.	0.495
**SCF3—Applicability of safety rules and safe work practices (SR&WP)**		
	(Mean = 2.42, Eigenvalue = 1.771, Variance = 7.379%, Cronbach’s coefficient alpha = 0.712)	
SC4	Some health & safety rules/procedures do not reflect how the job is to be done.	0.629
SC11	Some health & safety rules or procedures are difficult to follow as they are either too complex or not practical.	0.775
SC17	Sometimes it is necessary to take risks to get the job done within given time.	0.648
SC23	Some health & safety procedures are too stringent in relation to the associated risks.	0.587
SC29	Some jobs here are difficult to do safely due to physical conditions on site.	0.695
**SCF4—Safety consciousness and responsibility (SC&R)**		
	(Mean = 4.08, Eigenvalue = 1.449, Variance = 6.037%, Cronbach’s coefficient alpha = 0.648)	
SC19	I am very clear about my responsibilities for health & safety.	0.652
SC26	Work Health & safety is not my concern—it is not my responsibility.	0.810
SC28	Regular safety inspections are very helpful to improve the health & safety of workers.	0.730

Note: SC statements and SC factors are reported in detail in Zahoor et al. [36]. Rotation method: Promax with Kaiser Normalization; Rotation converged in 6 iterations. Overall mean of SC = 3.086, Cumulative variance = 56.18%, Cronbach’s coefficient alpha = 0.885.

**Table 2:** Principal component analysis to obtain SP indicators using calibration sub-sample.

Item No.	Statement	Factor Loading	Communalities	Mean	Cronbach’s Alpha
**Safety compliance (COMP)** (Eigenvalue = 3.332, Variance = 33.321%)				*3.281*	*0.921*
COMP1	You follow all of the safety procedures for the jobs that you perform.	0.891	0.849	3.585	
COMP2	Your co-workers (working in your team) follow all the safety procedures for the jobs that they perform.	0.956	0.921	3.246	
COMP3	All the workers in your company follow the safety procedures for the jobs that they perform.	0.929	0.836	3.011	
**Safety participation (PART)** (Eigenvalue = 2.384, Variance = 23.845%)				*3.374*	*0.87*
PART1	You always promote safety programmes at your workplace. (*e.g., always convincing the co-workers about the importance of safety compliance for our well-being*)	0.87	0.759	3.624	
PART2	How frequent do you put in extra effort to improve safety of the workplace? (*e.g., reminding the co-workers about safety procedures, reporting all incidents, looking for hazards*)	0.903	0.828	3.455	
PART3	How frequent do you voluntarily carry out tasks or activities that help to improve workplace safety? (*e.g., attending safety meetings, giving suggestions for improvements, receiving safety training voluntarily, and assisting the co-workers in safety compliance*)	0.885	0.787	3.042	
**Number of self-reported accidents/injuries and near-misses in past 12 months (ACC)** (Eigenvalue = 1.68, Variance = 16.802%)				*1.694*	*0.732 ^#^*
ACC1	How many times have you exposed to a near-miss incident of any kind at work?	0.539	0.345	2.338	
ACC2	How many times have you suffered from an accident/injury of any kind at work, but did NOT require absence from work?	0.809	0.643	1.699	
ACC3	How many times have you suffered from an accident/injury, which required absence from work NOT exceeding three consecutive days?	0.876	0.755	1.427	
ACC4	How many times have you suffered from an accident/injury, which required absence from work exceeding three consecutive days?	0.809	0.675	1.309	
Overall SP				2.674	0.68 ^#^
Cumulative % of variance		73.968			

Note: Rotation method: Promax with Kaiser Normalization; Rotation converged in 5 iterations. ^#^ After deleting ACC1, Cronbach’s alpha for ACC changed from 0.732 to 0.79, and for overall SP from 0.68 to 0.703, whereas mean value of ACC changed from 1.694 to 1.479, and for SP from 2.674 to 2.711. Similarly, cumulative % of variance improved from 73.968% to 79.566%.

**Table 3:** Demographic characteristics of the respondents.

Characteristics	Total (*N* = 426)	Characteristics	Total (*N* = 426)
***Age (years)***		***Education level***	
20 or below	93 (21.83%)	Below primary	21 (4.93%)
21–30	105 (24.65%)	Primary	32 (7.51%)
31–40	94 (22.06%)	Middle	41 (9.62%)
41–50	79 (18.55%)	Secondary	17 (3.99%)
51–60	43 (10.09%)	Diploma	135 (31.69%)
61 or above	12 (2.82%)	Degree or higher	180 (42.25%)
***Working level***		***Type of employer/organization***	
Frontline worker	85 (19.95%)	Client/Owner	77 (18.08%)
Foreman	26 (6.1%)	Main contractor	88 (20.66%)
Supervisor	58 ^@^ (13.62%)	Subcontractor	133 (31.22%)
Site Engineer	82 (19.25%)	Consultant	86 (20.19%)
Construction manager	98 ^#^ (23%)	Academia	42 (9.86%)
Safety Official	77 ^&^ (18.08%)		
***Service with the current employer***		***Work experience in the CI***	
Less than 1 year	174 (40.85%)	Less than 5 years	133 (31.22%)
1–5 years	213 (50%)	6–10 years	81 (19.01%)
6–10 years	24 (5.63%)	11–15 years	106 (24.88%)
11–15 years	10 (2.35%)	16–20 years	68 (15.96%)
More than 15 years	5 (1.17%)	More than 20 years	38 (8.92%)

Note: The percentages may not add to 100 because of rounding errors. ^#^ 55 were construction managers, 26 were resident engineers and 17 were project managers; ^&^ 31 were safety officers and 46 were safety inspectors. ^@^ 37 were supervisors and 21 were surveyors.

**Table 4:** KMO and Bartlett tests for calibration Sub-sample.

Tests for Data Appropriateness for EFA		SC	SP
Kaiser-Meyer-Olkin (KMO) measure of sampling adequacy		0.848	0.721
Bartlett test of sphericity	Approximate Chi-square	2301.445	1166.757
	Degree of freedom	276	45
	Significance	0.001	0.001

**Table 5:** Means, standard deviations and correlations among latent variables of calibration sub-sample.

Construct	Mean	SD	SCF1	SCF2	SCF3	SCF4	COMP	PART
SCF1	2.833	7.559						
SCF2	3.460	4.648	0.628					
SCF3	2.420	3.039	0.392	−0.035				
SCF4	4.083	1.758	0.366	0.260	0.252			
COMP	3.281	2.867	0.307	0.428	0.058	0.254		
PART	3.374	3.087	0.227	0.291	0.086	0.333	0.290	
ACC	1.479	2.128	−0.185	−0.074	−0.173	−0.374	−0.194	−0.001

**Table 6:** Comparison of goodness-of-fit indices of SEM models for calibration and validation sub-samples.

Model-Fit Indices		Calibration Sub-Samples			Validation Sub-Sample Model	Acceptable Fit Indices
		Model-1a *(Including ACC1)*	Model-1b *(After Deleting ACC1)*	Final Model ^#^		
Parsimonious fit	Chi-sq/df	2.153	2.141	1.999	1.984	Less than 2
Absolute fit	RMSEA	0.074	0.073	0.069	0.068	Less than 0.08
	P-Close	0.001	0.001	0.001	0.001	Less than 0.05
	GFI	0.763	0.77	0.788	0.778	0.5 (acceptable) 1.0 (excellent)
	AGFI	0.729	0.736	0.753	0.742	
Incremental fit	CFI	0.825	0.835	0.858	0.872	

**^#^** Final model is obtained after deleting ACC1 and drawing the correlations among 8 error variables.

**Table 7:** Reliability and validity measures for calibration sub-sample.

Construct	CR	AVE	√AVE	ASV	MSV	SCF1	SCF2	SCF3	SCF4	COMP	PART
SCF1	0.905	0.519	0.72	0.144	0.394	Squared factor correlation (R^2^) obtained from correlation matrix					
SCF2	0.788	0.353	0.594	0.123	0.394	0.394					
SCF3	0.718	0.347	0.589	0.043	0.154	0.154	0.001				
SCF4	0.657	0.390	0.625	0.097	0.140	0.134	0.068	0.064			
COMP	0.927	0.810	0.899	0.078	0.183	0.094	0.183	0.003	0.065		
PART	0.872	0.694	0.833	0.056	0.111	0.052	0.085	0.007	0.111	0.084	
ACC	0.812	0.596	0.772	0.041	0.140	0.034	0.005	0.030	0.140	0.038	0.001
